# Mitochondrial evidence for multiple radiations in the evolutionary history of small apes

**DOI:** 10.1186/1471-2148-10-74

**Published:** 2010-03-12

**Authors:** Van Ngoc Thinh, Alan R Mootnick, Thomas Geissmann, Ming Li, Thomas Ziegler, Muhammad Agil, Pierre Moisson, Tilo Nadler, Lutz Walter, Christian Roos

**Affiliations:** 1 Primate Genetics Laboratory, German Primate Center, Kellnerweg 4, 37077 Göttingen, Germany; 2 Gibbon Conservation Center, PO Box 800249, Santa Clarita, CA 91380, USA; 3 Anthropological Institute, University Zurich-Irchel, Winterthurerstrasse 190, 8057 Zurich, Switzerland; 4 Laboratory of Animal Ecology and Conservation Biology, Institute of Zoology, Chinese Academy of Sciences, 1 Beichen West Road, Chaoyang District, Beijing 100101, PR China; 5 Siberut Conservation Programme, Reproductive Biology Unit, German Primate Center, Kellnerweg 4, 37077 Göttingen, Germany; 6 Department of Clinic, Reproduction and Pathology, Faculty of Veterinary Medicine, Bogor Agricultural University, Jl. Agatis, Kampus IPB Darmaga, 16680 Bogor, Indonesia; 7 Parc Zoologique et Botanique de Mulhouse, 51, rue du Jardin Zoologique, 68100 Mulhouse, France; 8 Frankfurt Zoological Society, Endangered Primate Rescue Center, Cuc Phuong National Park, Nho Quan District, Ninh Binh Province, Vietnam; 9 Gene Bank of Primates, German Primate Center, Kellnerweg 4, 37077 Göttingen, Germany

## Abstract

**Background:**

Gibbons or small apes inhabit tropical and subtropical rain forests in Southeast Asia and adjacent regions, and are, next to great apes, our closest living relatives. With up to 16 species, gibbons form the most diverse group of living hominoids, but the number of taxa, their phylogenetic relationships and their phylogeography is controversial. To further the discussion of these issues we analyzed the complete mitochondrial cytochrome b gene from 85 individuals representing all gibbon species, including most subspecies.

**Results:**

Based on phylogenetic tree reconstructions, several monophyletic clades were detected, corresponding to genera, species and subspecies. A significantly supported branching pattern was obtained for members of the genus *Nomascus *but not for the genus *Hylobates*. The phylogenetic relationships among the four genera were also not well resolved. Nevertheless, the new data permitted the estimation of divergence ages for all taxa for the first time and showed that most lineages emerged during four short time periods. In the first, between ~6.7 and ~8.3 mya, the four gibbon genera diverged from each other. In the second (~3.0 - ~3.9 mya) and in the third period (~1.3 - ~1.8 mya), *Hylobates *and *Hoolock *differentiated. Finally, between ~0.5 and ~1.1 mya, *Hylobates lar *diverged into subspecies. In contrast, differentiation of *Nomascus *into species and subspecies was a continuous and prolonged process lasting from ~4.2 until ~0.4 mya.

**Conclusions:**

Although relationships among gibbon taxa on various levels remain unresolved, the present study provides a more complete view of the evolutionary and biogeographic history of the hylobatid family, and a more solid genetic basis for the taxonomic classification of the surviving taxa. We also show that mtDNA constitutes a useful marker for the accurate identification of individual gibbons, a tool which is urgently required to locate hunting hotspots and select individuals for captive breeding programs. Further studies including nuclear sequence data are necessary to completely understand the phylogeny and phylogeography of gibbons.

## Background

**Figure 1:**
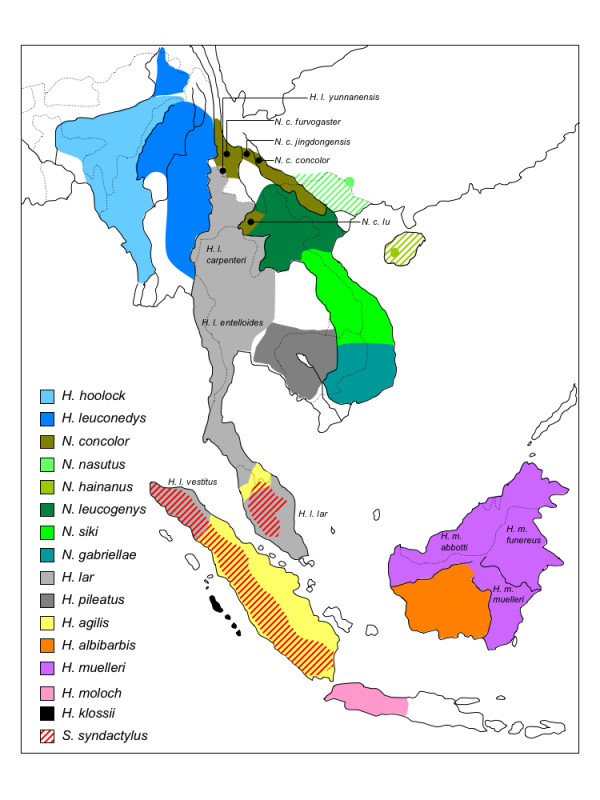
**Geographical distribution of gibbons based on **[2,5,23,41]. Dotted and solid lines indicate country borders and major rivers, respectively. Historical distribution of *N. hainanus *and *N. nasutus *is hatched.

Gibbons, family Hylobatidae, are small arboreal apes, which inhabit tropical and subtropical rainforests of Southeast Asia and adjacent regions (Figure 1). Together with humans and great apes, they belong to the primate superfamily Hominoidea [1-4]. Among hominoids, gibbons were the first to branch off and they display a set of morphological and behavioural characteristics distinctly different from great apes and humans [1,5,6]. Most prominent in this respect is the predominantly monogamous life style, their territorial calls, and the typical brachiating locomotion [1,4-7]. Due to their extensive karyotypic diversity [8-11], gibbons provide an excellent model organism to study chromosomal rearrangements and, hence, to better understand human diseases caused by such alterations.

Although in several aspects unique among primates and with up to 16 species the most diverse group of apes, gibbons are still in the shadow of great apes in respect of scientific studies, conservation efforts and public awareness. However, many gibbon species are on the brink of extinction and most of them are classified as "Endangered" or even "Critically Endangered" [12]. With approximately 20 individuals left in its native habitat, the Hainan gibbon (*Nomascus hainanus*) is the rarest primate in the world [6,13,14]. Responsible for this critical situation is habitat loss and hunting, which both have seriously reduced gibbon populations throughout their range [15,16]. Hence, much more attention has to be drawn on the gibbons' situation and extensive conservation actions are urgently required to save them from extinction [16].

While gibbons are widely considered to form a monophyletic clade, there is no consensus about the phylogeny and taxonomy within the family. Although various studies based on morphology, behaviour, vocalisation, protein electrophoresis, karyotyping and DNA sequencing were conducted [3-5,7,17-35], neither a congruent phylogeny nor a consistent taxonomic classification was obtained. Moreover, incomplete taxon sampling as well as misidentified specimens resulted in only fragmentary or even false conclusions. Accordingly, the classification of gibbon taxa at various taxonomic levels as well as their phylogenetic relationships remain disputed and a consensus is far from being available.

For example, in early studies, small apes were divided into two genera, with one (*Symphalangus*) including the siamang, and the other (*Hylobates*) all the remaining species [17,36]. Later on, the family was split into four major clades, which were recognized as subgenera [2,5,21] and eventually as genera [4,16,29,37,38]. This division is now widely accepted and takes into account the fact that species within each of the four major clades share a number of characteristics, most importantly a distinctive diploid chromosome number: *Hoolock *(2n = 38), *Hylobates *(2n = 44), *Symphalangus *(2n = 50) and *Nomascus *(2n = 52) [8]. Similarly, the number of species and subspecies is a matter of debate as well. While *Symphalangus *is consistently regarded as monotypic, the two *Hoolock *subspecies were recently elevated to species [38]. In *Nomascus *originally only one species was recognized [17,18,20,39], but in current classifications four to six species were suggested [2,4,12,16,34]. In contrast, the genus *Hylobates *already comprised at least four species in early classifications [17,39], but recent studies proposed six or seven species [2,4,16]. Due to this incongruence we follow the most recent classification of the IUCN Red List [12] with a total of 16 gibbon species (Table 1).

**Table 1:** Common names, IUCN classification and proposed classification of gibbons.

Common name	**IUCN classification **[12]	Proposed classification
Kloss' s gibbon	*Hylobates klossii*	*Hylobates klossii*
Eastern Müller's Bornean gibbon	*Hylobates muelleri muelleri*	Hylobates muelleri*
Northern Müller's Bornean gibbon	*Hylobates muelleri funereus*	Hylobates funereus*
Abbott's Müller's Bornean gibbon	*Hylobates muelleri abbotti*	Hylobates abbotti*
Agile gibbon	*Hylobates agilis*	Hylobates agilis*
Bornean white-bearded gibbon	*Hylobates albibarbis*	*Hylobates albibarbis*
Malayan lar gibbon	*Hylobates lar lar*	Hylobates lar lar*
Sumatran lar gibbon	*Hylobates lar vestitus*	Hylobates lar vestitus*
Mainland lar gibbon	*Hylobates lar entelloides*	Hylobates lar entelloides*
Carpenter's lar gibbon	*Hylobates lar carpenteri*	Hylobates lar carpenteri*
Yunnan lar gibbon	*Hylobates lar yunnanensis*	Hylobates lar yunnanensis*
Silvery Javan gibbon	*Hylobates moloch*	Hylobates moloch*
Pileated gibbon	*Hylobates pileatus*	*Hylobates pileatus*
Western hoolock gibbon	*Hoolock hoolock*	*Hoolock hoolock*
Eastern hoolock gibbon	*Hoolock leuconedys*	*Hoolock leuconedys*
Siamang	*Symphalangus syndactylus*	Symphalangus syndactylus*
Hainan gibbon	*Nomascus hainanus*	*Nomascus hainanus*
Cao-vit crested gibbon	*Nomascus nasutus*	*Nomascus nasutus*
Black crested gibbon	*Nomascus concolor concolor*	Nomascus concolor concolor*
West Yunnan black crested gibbon	*Nomascus concolor furvogaster*	Nomascus concolor concolor*
Central Yunnan black crested gibbon	*Nomascus concolor jingdongensis*	Nomascus concolor concolor*
Laotian black crested gibbon	*Nomascus concolor lu*	Nomascus concolor lu*
Northern white-cheeked gibbon	*Nomascus leucogenys*	Nomascus leucogenys*
Southern white-cheeked gibbon	*Nomascus siki*	Nomascus siki*
Red-cheeked gibbon	*Nomascus gabriellae*	*Nomascus gabriellae*

	**16 species, 12 subspecies**	**18 species, 7 subspecies**

*further research required

In the present study, we analyse the complete mitochondrial cytochrome b (cytb) gene from 85 individuals, which represent all gibbon genera and species, and most subspecies. Based on our data, we are able to 1) provide the most complete phylogeny of gibbons on all taxonomic levels, 2) estimate divergence times between lineages, 3) establish a reliable classification, 4) elucidate gibbon phylogeography, and 5) provide a tool for the species identification of gibbon individuals.

## Results

From all 85 gibbons, we successfully generated sequences of the complete mitochondrial cytb gene (1,140 bp). A contamination of our dataset with nuclear pseudogenes (numts) can be regarded as minimal, because no multiple amplifications of different copies were detected by direct sequencing. All sequences were correctly transcribed, and identical sequences were obtained for the same individual in cases where different material types were available. Moreover, no inconsistent positions were detected in alignments, which were assembled from overlapping sequences. Cross-contamination between individuals can be excluded as well, since all negative controls revealed no amplifications and randomly repeated PCRs for the same individual produced identical sequences.

Among the 85 individual gibbons studied, no identical haplotypes were detected. The cytb alignment comprising solely gibbons was characterized by 429 variable sites, of which 374 were parsimony-informative. In the complete alignment, which additionally contained great ape, human and hamadryas baboon representatives, we observed 565 variable sites, of which 462 were parsimony-informative.

**Figure 2:**
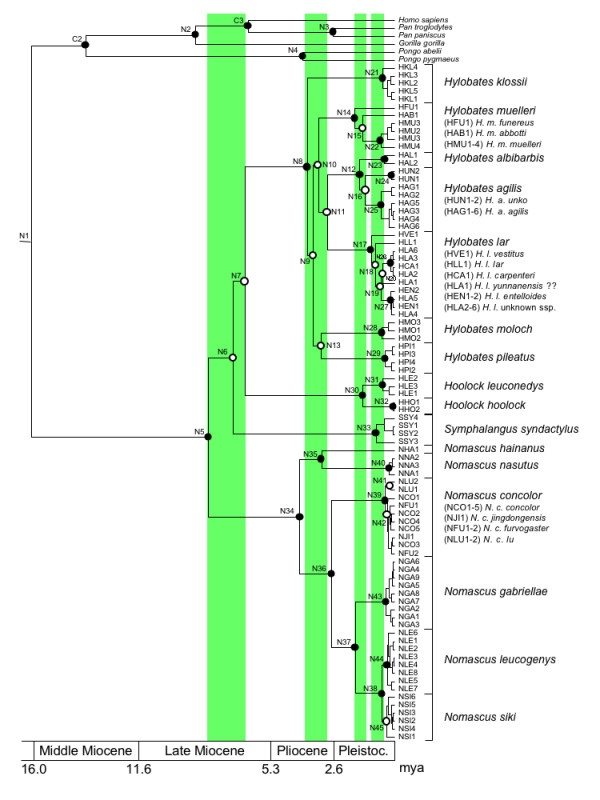
**Ultrametric tree showing phylogenetic relationships and estimated divergence ages among studied gibbon individuals based on complete mitochondrial cytb sequence data**. For individual codes see Additional File 1. Circles indicate bootstrap or posterior probability values (filled circles: >90%, >0.95, open circles: <70%, <0.80). Nodes of interest are arbitrarily numbered (N1-N45). C2 and C3 refer to two of the three nodes used for calibration (C1 not shown). Light green bars indicate the four radiations. A geological time scale is given below. Full details of age estimates and node supports are presented in Table 2.

Phylogenetic tree reconstructions based on maximum-parsimony (MP), neighbor-joining (NJ), maximum-likelihood (ML) and Bayesian algorithms revealed various strongly supported clades, which corresponded to genera, species and subspecies (Figure 2). All algorithms led to identical tree topologies, although several branching patterns gained only weak support. According to our reconstructions, hominoids diverged into a clade consisting of gibbons, and another with great apes and human. Among the latter, *Pongo *split off first, followed by *Gorilla*, before finally *Pan *and *Homo *diverged. Within gibbons, a basal position of *Nomascus *and a sister grouping of *Hylobates *and *Hoolock *was indicated, but support for this branching pattern was relatively low (Table 2). Similarly, with the exception of a strongly supported *H. agilis + H. albibarbis *clade, also the relationships among the species of *Hylobates *were not well resolved. However, at least species monophylies were clearly confirmed, though a common origin of *H. agilis *was only weakly supported. The relationships among the subspecies of *H. muelleri *and *H. lar *were less resolved. In *Hoolock*, the two species *H. hoolock *and *H. leuconedys *clearly segregated into two distinct clades. Within *Nomascus*, relationships among species were completely resolved, suggesting a *N. hainanus *+ *N. nasutus *clade as sister lineage to the remaining species. Among them, *N. concolor *branched off first, followed by the divergence of *N. gabriellae *and *N. leucogenys/N. siki*. The monophyly of *N. leucogenys *was significantly supported, but evidence for a common origin of *N. siki *individuals was not obtained. Within *N. concolor*, specimens identified as *N. concolor lu *formed a distinct clade, while the remaining subspecies clustered together without further subdivision. However, support for a reciprocal monophyly of both clades was relatively low.

**Table 2:** Support values and Bayesian divergence date estimates (in mya)*.

Node	Support values**	Divergence	Mean (95% CI)
C1		*Papio - *Hominoidea	24.04 (22.01-26.08)
N1		Hylobatidae - Hominidae	16.26 (14.69-18.16)
C2	96/92/92/0.99	*Pongo - Gorilla/Pan/Homo*	13.83 (13.28-14.41)
N2	91/93/98/1.0	*Gorilla - Pan/Homo*	8.90 (7.58-10.22)
C3	97/96/91/1.0	*Pan - Homo*	6.56 (6.01-7.08)
N3	100/91/97/0.99	*Pan troglodytes - P. paniscus*	2.74 (2.03-3.51)
N4	100/98/96/0.99	*Pongo pygmaeus - P. abelii*	4.12 (3.14-5.13)
N5	100/100/100/1.0	*Nomascus - Symphalangus/Hoolock/Hylobates*	8.34 (7.14-9.68)
N6	56/69/67/0.78	*Symphalangus - Hoolock/Hylobates*	7.22 (5.99-8.44)
N7	65/54/54/0.71	*Hoolock - Hylobates*	6.69 (5.56-7.88)
N8	100/93/94/0.99	*Hylobates klossii - H. pileatus/H. moloch/H. agilis/H. albibarbis/H. lar/H. muelleri*	3.91 (3.25-4.59)
N9	<50/68/<50/<0.50	*H. pileatus/H. moloch - H. agilis/H. albibarbis/H. lar/H. muelleri*	3.65 (3.05-4.25)
N10	<50/<50/<50/0.62	*H. muelleri - H. agilis/H. albibarbis/H. lar*	3.40 (2.81-3.99)
N11	<50/53/<50/0.69	*H. agilis/H. albibarbis - H. lar*	3.02 (2.43-3.60)
N12	100/99/100/1.0	*H. agilis - H. albibarbis*	1.56 (1.19-1.98)
N13	<50/52/<50/<0.50	*H. pileatus - H. moloch*	3.29 (2.64-3.97)
N14	96/96/98/1.0	*H. muelleri funereus - H. m. abbotti/H. m. muelleri*	1.78 (1.33-2.25)
N15	56/57/<50/<0.50	*H. muelleri abbotti - H. m. muelleri*	1.42 (1.02-1.81)
N16	63/<50/67/0.79	*H. agilis agilis - H. a. unko*	1.30 (0.95-1.68)
N17	100/100/99/1.0	*H. lar vestitus - H. l. lar/H. l. entelloides/H. l. carpenteri/H. l. yunnanensis*	1.05 (0.75-1.35)
N18	<50/<50/50/0.76	*H. l. lar - H. entelloides/H. l. carpenteri/H. l. yunnanensis*	0.86 (0.60-1.13)
N19	<50/63/65/0.79	*H. l. entelloides - H. l. carpenteri/H. l. yunnanensis*	0.62 (0.41-0.83)
N20	<50/66/66/0.78	*H. l. carpenteri - H. l. yunnanensis*	0.52 (0.32-0.71)
N21	100/100/99/1.0	MRCA *H. klossii*	0.53 (0.29-0.81)
N22	99/96/97/1.0	MRCA *H. muelleri muelleri*	0.62 (0.38-0.88)
N23	100/100/100/1.0	MRCA *H. albibarbis*	0.44 (0.22-0.68)
N24	100/100/100/1.0	MRCA *H. agilis unko*	0.13 (0.02-0.25)
N25	99/96/94/1.0	MRCA *H. agilis agilis*	0.61 (0.36-0.89)
N26	95/98/92/1.0	MRCA *H. lar carpenteri*	0.17 (0.05-0.28)
N27	96/94/96/1.0	MRCA *H. lar entelloides*	0.18 (0.07-0.31)
N28	100/100/94/1.0	MRCA *H. pileatus*	0.41 (0.21-0.64)
N29	100/100/100/1.0	MRCA *H. moloch*	0.56 (0.30-0.84)
N30	100/100/100/1.0	*Hoolock hoolock - H. leuconedys*	1.42 (0.97-1.90)
N31	99/95/93/0.96	MRCA *H. leuconedys*	0.51 (0.28-0.80)
N32	100/100/100/1.0	MRCA *H. hoolock*	0.07 (0.00-0.17)
N33	100/99/99/1.0	MRCA *Symphalangus syndactylus*	0.83 (0.51-1.18)
N34	100/100/99/1.0	*Nomascus hainanus/N. nasutus - N. concolor/N. gabriellae/N. leucogenys/N. siki*	4.24 (3.46-5.06)
N35	91/92/92/0.99	*N. hainanus - N. nasutus*	3.25 (2.49-3.99)
N36	94/91/96/1.0	*N. concolor - N. gabriellae/N. leucogenys/N. siki*	2.83 (2.21-3.50)
N37	96/92/98/1.0	*N. gabriellae - N. leucogenys/N. siki*	1.74 (1.28-2.22)
N38	100/99/93/1.0	*N. leucogenys - N. siki*	0.55 (0.35-0.77)
N39	100/100/100/1.0	*N. concolor lu - N. c. concolor/N. c. furvogaster/N. c. jingdongensis*	0.43 (0.25-0.63)
N40	100/100/99/1.0	MRCA *N. nasutus*	0.23 (0.08-0.39)
N41	<50/<50/67/0.75	MRCA *N. concolor lu*	0.19 (0.05-0.35)
N42	59/<50/<50/<0.50	MRCA *N. concolor concolor/N. c. furvogaster/N. jingdongensis*	0.32 (0.19-0.48)
N43	100/100/98/1.0	MRCA *N. gabriellae*	0.39 (0.21-0.57)
N44	92/91/98/1.0	MRCA *N. leucogenys*	0.33 (0.18-0.47)
N45	<50/<50/<50/0.58	MRCA *N. siki*	0.38 (0.18-0.55)

*Means and 95% credibility intervals (CI) are given for 48 nodes (see also Figure 2). Nodes used as calibrations are labelled with a "C", all others with an "N". MRCA denotes the most recent common ancestor. C1 not shown in Figure 2. **Support values as obtained from MP, NJ, ML and Bayesian reconstructions, respectively.

Based on divergence age estimates, gibbons separated from great apes and humans 16.26 million years ago (mya) (for 95% credibility intervals see Table 2). Within hominids, *Pongo *branched off first (13.83 mya), followed by *Gorilla *(8.90 mya), before finally *Homo *and *Pan *diverged from each other (6.56 mya). The differentiation of *Pongo *and *Pan *into species occurred 4.12 and 2.74 mya, respectively. In an initial radiation, gibbons diverged within a relative short time period of only 1.65 million years (6.69-8.34 mya) into four genera. Within *Hylobates*, most species diverged from each other between 3.02 and 3.90 mya. The only exception was the separation of *H. albibarbis *from *H. agilis *1.56 mya, which was in the time frame of subspecies splits within *H. muelleri *(1.42-1.78 mya). Differentiation of *H. lar *into subspecies occurred even later (0.52-1.05 mya). The two *Hoolock *species diverged 1.42 mya from each other. In *Nomascus*, differentiation into species took place over a longer time period, lasting from 4.24 until 0.55 mya. The most recent species divergence within *Nomascus *occurred between *N. siki *and *N. leucogenys *(0.55 mya), which was in a similar range as the separation of *N. concolor lu *from the other *N. concolor *subspecies (0.43 mya).

## Discussion

By analysing all species and most subspecies, the present study provides the most complete view into the evolutionary history of the gibbon family. However, as in earlier molecular studies on gibbons [26-35], relationships on various taxonomic levels are less resolved and partially contradict earlier findings. While the herein depicted branching pattern among genera is identical with that found in earlier studies using also cytb [32] or D-loop [29] sequences, it differs from another cytb-based study [28] in placing *Nomascus *and not *Symphalangus *as most basal genus. Studies based on mitochondrial ND3-ND4 sequences [31] or chromosomal rearrangements [8] suggest *Hoolock *as most ancestral lineage, and *Nomascus *together with either *Hylobates *[31] or *Symphalangus *[8] as the most recently diverged genera. For *Hylobates*, our data indicate a basal position of *H. klossii*, and a further division into a clade consisting of *H. lar, H. muelleri, H. agilis *and *H. albibarbis*, and another one with *H. moloch *and *H. pileatus*. Various branching patterns among *Hylobates *species are proposed [27,31,32,35], which all differ from our one, but respective support values are similarly low as in our study. In contrast, the relationships found among species of the genus *Nomascus *are well resolved and identical with that suggested by [30,31,33,34].

According to our and earlier data, relationships among gibbon genera and *Hylobates *species remain disputed, which most likely can be explained by the separation of respective lineages within relative short time periods. This becomes even more obvious when considering estimated divergence ages, which fall into four temporal windows. In the first, between ~6.7 and ~8.3 mya, the four gibbon genera originated. In a second radiation, between ~3.0 and ~3.9 mya, *Hylobates *split into various species, and in a third burst, between ~1.3 and ~1.8 mya, *H. muelleri*, the *H. agilis *+ *H. albibarbis *clade and *Hoolock *further differentiated. Finally, in a fourth radiation, between ~0.5 and ~1.1 mya, *H. lar *diverged into subspecies. In contrast, speciation in *Nomascus *was a continuous process, lasting from 4.24 until 0.55 mya.

### Taxonomic implications

Our data show that mitochondrial DNA (mtDNA) provides a powerful tool for the identification and taxonomic classification of gibbons, because taxa form strongly supported monophyletic clades, or at least appear to form distinct lineages in those cases where only one individual per taxon was tested. Moreover, most differentiation events fall into four temporal periods, which allow a hierarchical ranking as proposed by Goodman et al. [40], though the threshold for the recognition of a certain taxonomic unit whether genus, species, or subspecies remains disputed. Hence, to provide a more reliable classification, we compare divergence ages among gibbon lineages with those among other Asian primates and hominids.

Accordingly and concordant with recent classifications [4,12,16,29,34,37,38,41], the four major gibbon lineages are proposed as distinct genera (Table 1), since they split from each other in a similar time range as did colobine genera [[42,43], Roos C, Zinner D, Schwarz C, Nash SD, Xing J, Batzer MA, Leendertz FH, Ziegler T, Perwitasari-Farajallah D, Nadler T, Walter L, Osterholz M: Nuclear versus mitochondrial DNA: evidence for hybridization in colobine monkeys, submitted] or African great apes and human [40,42]. Most species of *Hylobates *and *Nomascus *emerged in or around the second radiation, which is on the same time scale as species splits within *Pongo *and *Pan*, and the separation of species groups within *Macaca *[44,45] and *Trachypithecus *[46]. Thus, taxa originating in this time period should be recognized as distinct species (*H. moloch, H. pileatus, H. klossii, H. lar, H. muelleri, H. agilis/H. albibarbis, H. hoolock/H. leuconedys, N. nasutus, N. hainanus, N. concolor, N. gabriellae/N. leucogenys/N. siki*), and might be even classified as species groups. Further differentiation events among gibbons occurred in the third time period, which is in a similar window as several speciation events within macaques [44,45]. Accordingly, *H. leuconedys *and *H. albibarbis *should be separated from *H. hoolock *and *H. agilis *on species level, respectively, and the three subspecies of *H. muelleri *could be considered for elevation to species level. Moreover, *H. agilis *is divided into two clades, which refer to individuals identified by pelage coloration as *H. agilis agilis *and *H. agilis unko*. However, in a recent work based on a larger number of individuals a reciprocal monophyly of both lineages is doubted [47], and, hence, we provisionally recognize *H. agilis *as monotypic. For *H. lar*, only a few unambiguously identified specimens were available for our study, but these represent at least four of the five recognized subspecies, while the identity of the putative *H. lar yunnanensis *individual remains uncertain. Based on our data, *H. lar *subspecies form distinct lineages, which diverged relative recently. We provisionally accept all five subspecies, though ongoing studies might reject some or all of them. For *N. concolor*, our data indicate a separation of *N. concolor lu *from the remaining subspecies, which form a clade without further subdivision into taxa. Hence and concordant with Monda et al. [33] and Roos et al. [34], we provisionally classify *N. concolor furvogaster *and *N. concolor jingdongensis *as synonyms of *N. concolor concolor*, while we feel *N. concolor lu *is a separate subspecies. We further separate *N. gabriellae *from *N. siki/N. leucogenys *on species level, while it is questionable whether the latter two should be recognized as species or subspecies. Our study reveals a split between both taxa just 0.55 mya, which is in a similar range as the subspecies differentiation within *H. lar *or *N. concolor*. Hence, a separation of both taxa only on subspecies level would be indicated. However, both taxa show slight differences in vocalisation and facial colouration [4,5,15], and Carbone et al. [48] found a chromosomal inversion unique to *N. leucogenys*. Accordingly, we follow here the current view and recognize *N. leucogenys *and *N. siki *as distinct species. In summary, we recognize four gibbon genera with 18 species and seven subspecies (Table 1).

### Biogeographic implications

Multiple radiations in the evolutionary history of gibbons suggest a complicated biogeographic pattern leading to the current distribution of gibbon taxa. Since gibbons are arboreal [7,39], radiations most likely were correlated with expanding forest habitats. In fact, the complete range of gibbons experienced complex geographical and environmental changes during the last ten million years. Notably, in the late Miocene as well as in the Plio- and Pleistocene, a series of dramatic climatic changes influenced the geography and vegetation in the region, leading to shifts in the extension and distribution of different habitat types [49-54]. In particular, periods of maximum glaciation might have reduced rainforest cover, resulting in the appearance of more open and deciduous vegetation types in many parts of the region [[52-57], but see [58]]. Moreover, due to the alternately falling and rising sea water levels during the several glacial and interglacial periods [59-64], connections and separations of landmasses were common, and repeated migration between islands and today's mainland was possible [65-67].

By combining the available information, we develop the following dispersal scenario for gibbons, which is in general agreement with that proposed by Chatterjee [32,68], Harrison et al. [69], and Jablonski and Chaplin [70], but which differs substantially from them in some aspects. Accordingly, gibbons most likely originated on the Asian mainland, because all four gibbon genera occur there. Specifically, the Hengduan mountains in the border region of today's Burma, India and China might have been a possible diversification hotspot [71,72]. In the region, all the larger Southeast Asian rivers (Mekong, Salween, Yangtze) rise, which are all well known as barriers for arboreal primates [54]. Although these rivers changed their courses several times, their upper reaches in the Hengduan mountains exist at least since the early Miocene [73]. Recently, the Hengduan mountains were also proposed as a region of differentiation for colobine monkeys, and, most interestingly, respective splitting events occurred on a similar time scale as in gibbons [Roos C, Zinner D, Schwarz C, Nash SD, Xing J, Batzer MA, Leendertz FH, Ziegler T, Perwitasari-Farajallah D, Nadler T, Walter L, Osterholz M: Nuclear versus mitochondrial DNA: evidence for hybridization in colobine monkeys, submitted]. In fact, in the late Miocene, widely distributed rain forest habitats promoted range extension for arboreal primates [50,54]. Accordingly, in the late Miocene, *Nomascus *invaded the region east of the Mekong, *Hoolock *entered the region west of the Salween, and *Hylobates *and *Symphalangus *migrated into the area in-between and later on into Sundaland.

*Hylobates *successfully colonized large parts of Sundaland, but also survived on the Asian mainland. Shortly after its arrival in Sundaland in the Pliocene, populations on the Asian mainland, the Malay peninsula, Sumatra, Borneo, Java and the Mentawai archipelago became isolated. At the same time, various species groups of the genera *Macaca *and *Trachypithecus *diverged [44-46], indicating dramatic environmental changes. In fact, this time period was characterized by global warming and sea levels similar to today [54,61-63], which prevented migration between landmasses and, thus, promoted speciation due to vicariance. Whether *Symphalangus *experienced a similar range expansion in Sundaland like *Hylobates*, remains questionable. Today the genus appears only on Sumatra and the Malay peninsula, and fossil data provide only evidence for its historical occurrence on Java and Sumatra [69]. In the early Pleistocene, further differentiation in *Hylobates *occurred on Borneo and Sumatra, and in *Hoolock *on the mainland which is on a similar time scale when macaque species diverged [44,45], and which might has been triggered by the shrinking of forest habitats due to cold phases [[74], but see [58]]. Notably, *H. albibarbis *is mitochondrially closer related to Sumatran *H. agilis *than to the other Bornean gibbons, and acoustic, morphological and chromosomal data suggest an intermediate position [2,5,47,75]. Accordingly, *H. albibarbis *might be the product of an ancient hybridization event, in which proto-*H. agilis *invaded Borneo during sea level lowstands [61-64], and successfully reproduced with proto-*H. muelleri*. As we find mtDNA of proto-*H. agilis *in *H. albibarbis*, female introgression is the most likely hybridization scenario, which is in agreement with recent findings, that gibbon females disperse over longer distances than males [76]. Finally, in a last range expansion in the early to middle Pleistocene, *H. lar *colonized, starting from its Sumatran refuge, the Malaysian peninsular and mainland Southeast Asia [see also [70]].

In contrast to the biogeographic pattern found in *Hylobates *and to the scenario proposed by Chatterjee [32,68], for *Nomascus *not a radiation but a successive migration from North to South over a long time period becomes evident. Based on our data, *Nomascus *originated in the border region of Vietnam and China in the early Pliocene and it took to the early Pleistocene until the genus reached the southern extend of its current distribution in southern Vietnam and Cambodia.

### Conservation implications

All gibbon species are on the brink of extinction and, with the exception of *H. leuconedys *(Vulnerable), are classified as "Endangered" or even "Critically Endangered" [12,16]. With approximately 20 individuals left in its native habitat, the Hainan gibbon (*N. hainanus*) is the rarest primate in the world [6,13,14], and the situation for its closest relative, the Cao-vit crested gibbon (*N. nasutus*) with approximately 100 individuals left [12,77], as well as for other gibbon species, the situation is alarming. Reasons for the decline of gibbons are manifold, but habitat loss due to forest clearance for agricultural use, oil palm or rubber plantations, gold mining, or charcoal and timber production, as well as illegal hunting for food and sport, and the trade for pets or medicine are major threats to wild gibbon populations [15,16].

To save gibbons from extinction, urgent actions are required to prevent ongoing habitat destruction and hunting, and to build up a viable gene pool in captivity for later release purposes. Specifically, to prevent or at least reduce hunting, hunting hotspots have to be identified. Therefore, it is crucial to confirm the taxon identity and if possible the geographical origin of confiscated gibbons or their remains. Similarly, to avoid artificial hybrids, only gibbons with clear taxon identity should be considered for reproduction in zoos or rescue centres. Finally, if captive gibbons are reintroduced into the wild, it has to be ascertained that these gibbons are pure individuals and of the same taxon as those, which naturally occur in the area they are to be released.

An accurate taxonomic identification of gibbons based on vocal data or pelage colouration is sometimes complicated [4,5]. In this respect, mtDNA analysis might be a promising tool. As shown in our study, gibbon taxa can be diagnosed through mtDNA, and, hence, a secure identification can easily be obtained. Yet since mtDNA is only maternally inherited, possible hybrids will not be detected in such analysis, so that additional markers should be studied as well.

## Conclusions

Due to a nearly complete taxon sampling, the present study provides the most comprehensive insights into the evolutionary and biogeographic history of the hylobatid family. Based on estimated divergence ages and unresolved relationships among gibbon taxa on various levels, several radiation-like splitting events are indicated, which suggest a complex biogeographic history of gibbons. Presumably, most of these differentiation events occurred in wave-like range expansions in Sundaland and the Asian mainland followed by vicariance effects, most likely caused by alternately shrinking and expanding rain forest habitats and by repeated separations and connections of landmasses. In contrast, in the region east of the Mekong river gibbons underwent a successive North-to-South migration. Our study also shows that mtDNA provides a solid platform for the taxonomic classification of gibbons and that mtDNA can be successfully applied to accurately identify the species affiliation of gibbon individuals, which is urgently required for conservation purposes. However, to completely understand the phylogeny and phylogeography of gibbons, to identify hybrids in captivity, or to trace possible ancient hybridization events as it might be indicated for *H. albibarbis*, further studies including extended mitochondrial as well as autosomal, X and Y chromosomal sequence data, are necessary.

## Methods

### Sample Collection

A total of 85 specimens representing all species and most subspecies of hylobatids were included in our study. Blood, tissue, faecal or hair samples were collected during field surveys, in zoos or rescue centres, or from museum specimens between 1995 and 2008 (Additional file 1). Blood and hair samples were taken during routine health checks by veterinarians. Tissue samples were obtained only from deceased animals. Taxon identity of individuals was ascertained by pelage coloration, morphology and if possible by vocalization and geographic origin. With the exception of some *H. lar *individuals for which subspecies identity could not be traced, only clearly identified specimens were included in our study. Fresh tissue or faecal samples were preserved in 80-90% ethanol and dry samples (tissue, museum skins, hair samples) were placed in plastic bags without any additive. Samples were stored at ambient temperature for up to six months before further processing.

### Laboratory Methods

Total genomic DNA was extracted with the DNeasy Blood & Tissue and QIAamp DNA Stool Mini kits from Qiagen. When hair follicle cells were used, up to three hairs were directly implemented into the PCR reaction. From high-quality DNA, the complete mitochondrial cytb gene was PCR-amplified in a single fragment with the primers 5'-AATGATATGAAAAACCATCGTTGTA-3' and 5'-TTCATTTCCGGCTTACAAGAC-3'. For low-quality DNA, extracted from faeces or museums material, two to seven overlapping PCR products were amplified with primers constructed on the basis of sequences from conspecifics (respective primers are available from the authors upon request). For all amplifications, wax-mediated hot-start PCRs were performed for 40 cycles, each with a denaturation step at 92°C for 1 min, annealing at 60°C for 1 min, and extension at 72°C for 0.5-1.5 min, followed by a final extension step at 72°C for 5 min. The results of the PCR amplifications were checked on 1% agarose gels. Subsequently, PCR products were cleaned with the Qiagen Gel Extraction kit and sequenced on an ABI 3130xl sequencer using the BigDye Cycle Sequencing kit. Sequences were assembled with Geneious v4.6.1 [78] and checked for their potential to be correctly transcribed. Gibbon haplotypes were deposited at GenBank and are available under the accession numbers GU321245-GU321329 (see also Additional file 1).

To prevent cross-species contaminations, laboratory procedures followed described standards [46]. To exclude contaminations of the dataset with numts, we mainly used material in which nuclear DNA is highly degraded (faeces, museum tissue) [79,80]. Moreover, the applied primers are known to amplify solely the mitochondrial copy of the gene in hylobatids [34], and for cross-validation purposes, for some specimens, sequences were generated using different material types (blood, faeces).

### Statistical Methods

For phylogenetic reconstructions, we expanded our dataset with orthologous sequences from various hominids (*Homo, Pan, Gorilla, Pongo*) and *Papio hamadryas*, which was used as outgroup. Phylogenetic trees were constructed with MP and NJ algorithms as implemented in PAUP v4.0b10 [81] as well as with ML and Bayesian algorithms, using the programs GARLI v0.951 [82] and MrBayes v3.1.2 [83,84]. For MP analysis, all characters were treated as unordered and equally weighted throughout. A heuristic search was performed with the maximum number of trees set to 100. For NJ and ML reconstructions, the optimal nucleotide substitution model (GTR + Γ) was chosen using Akaike information criterion (AIC) as implemented in MODELTEST v3.7 [85]. Relative support of internal nodes was performed by bootstrap analyses with 10,000 (MP, NJ) or 500 replications (ML). In GARLI, only the model specification settings were adjusted according to the dataset, while all other settings were left at their default value. ML majority-rule consensus trees were calculated in PAUP. For Bayesian reconstructions, the dataset was partitioned into codon positions, each with its own substitution model. We used four Markov Chain Monte Carlo (MCMC) chains with the default temperature of 0.1. Four repetitions were run for 10,000,000 generations with tree and parameter sampling occurring every 100 generations. The first 25% of samples were discarded as burnin, leaving 75,001 trees per run. Posterior probabilities for each split and a phylogram with mean branch lengths were calculated from the posterior density of trees.

To estimate divergence times, a Bayesian MCMC method, which employs a relaxed molecular clock approach [86], as implemented in BEAST v1.4.8 [87], was used. A relaxed lognormal model of lineage variation and a Yule prior for branching rates was assumed. The alignment was partitioned into codon positions, and the substitution model, rate heterogeneity and base frequencies were unlinked across codon positions. Optimal nucleotide substitution models were chosen using AIC in MODELTEST.

For calibrations we used the fossil-based divergence between *Homo *and *Pan*, which was dated at 6 - 7 mya [88-90], the separation of *Pongo *from the *Homo*/*Pan *lineage ~14 mya [91], and the divergence of hominoids and cercopithecoids ~23 mya [92,93]. Instead of hardbounded calibration points, we used the published dates as a normal distribution prior for the respective node. For the *Homo *- *Pan *divergence, this translates into a normal distribution with a mean of 6.5 mya and a standard deviation (SD) of 0.5 mya, for the separation of *Pongo *from the *Homo*/*Pan *clade into a mean of 14.0 mya and a SD of 1.0 mya, and for the hominoid - cercopithecoid divergence into a mean of 23 mya and a SD of 2 mya.

Since the estimation of phylogenetic relationships was not the main aim of this analysis, for the calculation an a-priori fixed tree topology as obtained from NJ reconstructions using the GTR + Γ model (Figure 2) was implemented. Four replicates were run for 10,000,000 generations with tree and parameter sampling occurring every 100 generations. The adequacy of a 10% burnin and convergence of all parameters were assessed by visual inspection of the trace of the parameters across generations using TRACER v1.4.1 [94]. Subsequently, the sampling distributions were combined (25% burnin) using the software LogCombiner v1.4.8, and a consensus chronogram with node height distribution was generated and visualized with TreeAnnotator v1.4.8 and FigTree v1.2.2 [95].

## Authors' contributions

VNT collected samples, did laboratory work, analysed the data, and wrote the paper. ARM, TG, LM, TZ, MA, PM, and TN collected samples and wrote the paper. LW analysed data, and wrote the paper. CR designed the study, collected samples, did laboratory work, analysed data, and wrote the paper. All authors read and approved the final manuscript.

## Supplementary Material

Additional file 1 Origin, material type, sample provider/collector and GenBank accession numbers of studied gibbon specimens. Click here for file
